# Evaluation of the Early-Mid-Term Results of Isolated Tibial Insert Exchange After Total Knee Arthroplasty

**DOI:** 10.7759/cureus.36943

**Published:** 2023-03-31

**Authors:** İbrahim Eke, Mehmet Akif Akcal

**Affiliations:** 1 Orthopedics and Traumatology, Antalya Atatürk Public Hospital, Antalya, TUR; 2 Orthopedics and Traumatology, Antalya Private Anadolu Hospital, Antalya, TUR

**Keywords:** western ontario and mcmaster universities arthritis index (womac), isolated tibial insert exchange, visual analogue scale (vas), tka (total knee arthoplasty), persistent knee pain, knee function

## Abstract

Objective: This study aimed to assess the early and mid-term results of patients who had undergone total knee arthroplasty (TKA) and then underwent an isolated tibial insert exchange due to tibial insert fracture and/or melting.

Methods: A retrospective study was conducted at the Orthopedics and Traumatology Clinic in a secondary-care public hospital in Türkiye on seven knees of six patients aged 65 years and above who underwent an isolated tibial insert exchange and were followed up for at least six months. Pain and functional assessments of the patients were made with the visual analog scale (VAS) and Western Ontario and McMaster Universities Osteoarthritis Index (WOMAC) performed at the last control before the treatment and at the final follow-up visit after the treatment.

Results: The median age of the patients was 70.5 years. The median length of time between the primary TKA and the isolated tibial insert exchange was 5.96 years. After isolated tibial insert exchange, the patients were followed for a median of 268 days and a mean of 414 days. The WOMAC pain, stiffness, function, and total indexes were median 15, 2, 52, and 68, respectively, before the treatment. In contrast, the final follow-up WOMAC pain, stiffness, function, and total indexes were median 3 (p = 0.01), 1 (p = 0.023), 12 (p = 0.018), and 15 (p = 0.018), respectively. It was observed that the median VAS, which was “9” preoperatively, showed a statistically significant improvement to become “2” in the postoperative period. A strong negative correlation was found between age and the amount of decline in the total score of the WOMAC pain scale (r = -0.780; p = 0.039). There was a powerful negative correlation between the body mass index (BMI) and the amount of decline in WOMAC pain scores (r = -0.889; p = 0.007). A strong negative correlation was found between the length of time passing between two surgical procedures and the amount of decline in the WOMAC pain score (r = -0.796; p = 0.032).

Conclusion: Individual patient factors and prosthetic conditions should be considered undoubtedly when determining the best revision strategy in TKA patients. In cases where the components are well-aligned and well-fixed, isolated tibial insert exchange is an alternative to revision TKA since it is less invasive and more cost-effective.

## Introduction

Osteoarthritis of the knee is defined as a set of changes involving articular cartilage damage, abnormal bone formation, reactive changes in the synovial membrane, and pathologic synovial fluid [1]. It has recently become an important public health issue, the frequency of which is increasing day by day, due to the aging population all over the world as well as in Türkiye, the increased prevalence of obesity, and the rapid world population growth. As a consequence, total knee arthroplasty (TKA) rates have increased, and orthopedic surgeons have gained more experience in this regard [2].

TKA is indicated for patients with end-stage degenerative and inflammatory knee osteoarthritis who do not respond to conservative treatment. All possible non-surgical treatments should be tried, and the TKA should be postponed as much as possible [3,4]. In general, the durability of the prosthesis in patients who have undergone a TKA ranges from about 15 to 25 years [5,6]. Nevertheless, knee replacements can unfortunately fail and require further revision for a variety of reasons, including loosening, infection, persistent pain, and instability [5]. Even due to the biophysical insufficiency of the prosthesis, the tibial insert may sometimes melt, and/or the concave shape may be deformed due to abrasion or breakage may occur, leading to the need for a revision surgery much earlier (five to six years). The relevant developments especially in modular knee prostheses over the years have allowed orthopedic surgeons to exchange the polyethylene tibial part at revision surgery rather than revising the tibial metal plate [7]. Therefore, unless there is loosening of the tibial metal implants/components in patients and as long as such components are well-aligned and well-fixed, using a tibial insert exchange may be considered as a treatment since it is much more cost-effective and less invasive for the patient, besides having a much lower risk of complications and a shorter hospitalization than a revision TKA. The reason is that such a revision is rather costly, and it can unfortunately lead to much worse outcomes than primary surgery [5,8,9].

The aim of this study was to assess the early and mid-term results of patients who had undergone TKA and then underwent isolated tibial insert exchange due to tibial insert fracture and/or melting.

## Materials and methods

Study design and participants

This retrospective study was conducted at the Orthopedics and Traumatology Clinic in a secondary-care public hospital in Türkiye on seven knees of six patients aged 65 years and above who had previously undergone a TKA but then underwent an isolated insert exchange due to tibial insert degeneration between 2019 and 2022 and were followed up for at least six months. The primary TKA of six knees of five patients and isolated tibial insert exchange of seven knees of six patients were performed by the first author of this study.

Data collection tools and clinical assessment

Demographic characteristics such as age and gender, comorbidities, clinical and radiographic features, as well as treatment and outcome data of the patients in the study sample, were collected from the electronic medical records and patients’ surgery notes.

The knees were selected for isolated tibial insert exchange when the surgeon believed the components were well-aligned and well-fixed based on examination and radiographs. None of the knees presented any problems in the preoperative period, concerning the range of motion, and there was no severe or very severe stiffness. Four knees of four patients who underwent an isolated tibial insert exchange had been suffering from a broken insert, and three knees of the remaining two patients already had a loss of concave shape due to insert meltdown, resulting in instability and abnormal movements in seven knees. Moreover, intraoperatively, all components were found to be well-aligned and well-fixed.

Before and after the surgery, the patients were assessed by the surgeon via clinical examination (pain, range of motion, and revision status), radiographs, and patient questionnaires at all visits. Pain and functional assessments of the patients included the visual analog scale (VAS) [10] and Western Ontario and McMaster Universities Osteoarthritis Index (WOMAC) [11], conducted before the treatment and the final follow-up visit after the treatment.

The isolated tibial insert exchange was defined as a failure if the patients underwent a revision for any reason (repeat isolated tibial insert exchange or other components revised) or if they had persistent or moderate to severe pain.

WOMAC Osteoarthritis Index

WOMAC osteoarthritis index is a valid and reliable index that is widely used for the assessment of patients with osteoarthritis. In this index, pain is assessed with five questions, stiffness with two questions, and functional level with 17 questions. For each question, the lowest score is “0” and the highest is “4,” and a higher score indicates a more negative result in terms of pain, stiffness, and functional aspects. The Turkish validity and reliability study of the index was performed by Tüzün et al. in 2005 [11].

Ethical approval

Before the study, necessary ethical approval was obtained from the Clinical Research Ethics Committee of the University of Health Sciences Antalya Training and Research Hospital. The study was conducted in accordance with the Declaration of Helsinki.

Statistical analysis

The normality assumption was controlled with the Shapiro-Wilk test. Categorical variables were given as frequencies (n) and percentages (%). Continuous variables were expressed as the median and interquartile range (25-75 percentile). The Wilcoxon signed-rank test was used to compare the preoperative and postoperative WOMAC and VAS scores. The Spearman correlation test was applied to determine the association among study parameters. Data were analyzed by using SPSS (Statistical Package for the Social Sciences) Statistics for Windows, version 23.0 (IBM Corp., Armonk, NY). A value of two-sided p < 0.05 was considered statistically significant.

## Results

The median age of the patients was 70.5 (68-75) years, and the median body mass index (BMI) was 30.15 (29.45-31.29) kg/m^2^. All patients had hypertension, with 83.3% of them having coronary artery disease and 33.3% having diabetes mellitus. The median length of time between the primary TKA and the isolated tibial insert exchange was 5.96 years. After the isolated tibial insert exchange, the patients were followed for a median of 268 days and a mean of 414 days. A total of four (57.1%) of the operated knees were right-sided, while three (42.9%) were left (Table 1).

**Table 1:** Patient characteristics

Variables	All patients (n = 6)
Age (years), median (IQR)	70.5 (68-75)
Height, median (IQR)	165.5 (162-167)
Weight, median (IQR)	81.5 (81-87)
BMI, median (IQR)	30.15 (29.45-31.29)
Comorbidities, n (%)	
Hypertension	6 (100)
Diabetes mellitus	2 (33.3)
Chronic obstructive pulmonary disease	1 (16.7)
Coronary artery disease	5 (83.3)
Hyperlipidemia	1 (16.7)
Cerebrovascular disease	1 (16.7)
Chronic renal failure	1 (16.7)
Time between two surgical procedures (years), median (IQR)	5.96 (4.83-7.33)
Follow-up time (days), median (IQR)	268 (186-374)

The WOMAC pain, stiffness, function, and total indexes were median 15, 2, 52, and 68, respectively, in the final follow-up visits of six patients before the treatment, whereas the final follow-up WOMAC pain, stiffness, function, and total indexes were median 3 (p = 0.01), 1 (p = 0.023), 12 (p = 0.018) and 15 (p = 0.018), respectively. It was observed that the VAS, which was “9” preoperatively, showed a statistically significant improvement to become “2” in the postoperative period (p = 0.016) (Table 2).

**Table 2:** Change in WOMAC and VAS scores (n = 7) WOMAC: Western Ontario and McMaster Universities Osteoarthritis Index; VAS: Visual analog scale. Wilcoxon signed-rank test was used.

Scores, median (IQR)	Pre-op	Post-op	p-value
WOMAC			
Pain	15 (14-16)	3 (3-4)	0.017
Stiffness	2 (1-3)	1 (0-1)	0.023
Function	52 (50-54)	12 (11-13)	0.018
Total	68 (68-71)	15 (15-17)	0.018
VAS	9 (8-9)	2 (1-2)	0.016

A strong negative correlation was found between age and the amount of decline in the total score of the WOMAC pain scale (r = -0.780; p = 0.039). There was a negative correlation between age and the amount of decline in the WOMAC pain scale and WOMAC stiffness scores, though not statistically significant (p > 0.05). There was a powerful negative correlation between BMI and the amount of decline in WOMAC pain scores (r = -0.889; p = 0.007). Despite the presence of a negative correlation between BMI and the amount of decline in WOMAC total scores, it was not statistically significant (p = 0.334). A strong negative correlation was found between the length of time passing between two surgical procedures and the amount of decline in WOMAC pain score (r = -0.796; p = 0.032) (Table 3).

**Table 3:** Correlation between the amount of change in scores and other variables (n = 7) WOMAC: Western Ontario and McMaster Universities Osteoarthritis Index. The Spearman correlation test was used.

	Age		BMI		Time between two surgical procedures	
Difference (before-after)	r	p	r	p	r	p
WOMAC						
Pain	-0.667	0.102	-0.889	0.007	-0.796	0.032
Stiffness	-0.442	0.320	-0.201	0.666	-0.302	0.511
Function	-0.273	0.554	0.200	0.667	0.109	0.816
Total	-0.780	0.039	-0.431	0.334	-0.266	0.564
VAS	-0.030	0.949	0.070	0.882	-0.169	0.717

In one patient, only seven months after the isolated tibial insert exchange, the insert was broken again requiring a revision TKA, and so, that patient was considered a failure. For the same patient, the time taken for a revision after the primary TKA was the highest (98 months), and BMI (31.29 kg/m^2^) was the second highest. In addition, this patient did not have any other comorbidity other than hypertension. In the six knees of the remaining five patients, no pain and stiffness nor any function-related and other complaints were reported in the postoperative final follow-up.

## Discussion

This study assessed the early and mid-term results of patients who had previously undergone a TKA with the diagnosis of knee osteoarthritis and later underwent an isolated tibial insert exchange for further treatment due to pain, inability to walk, or abnormal movements relatively. The median time between primary TKA and isolated tibial insert exchange was 5.96 years. The patients were followed for a median of 268 days after the procedure. Statistically significant improvement was observed in the pain, stiffness, and function in the WOMAC scale as well as the total WOMAC scores considering the changes in the control values just before the isolated tibial insert exchange and the final follow-up after the procedure in seven knees of six patients. A statistically significant improvement was also observed in the VAS scores. A strong negative correlation was found between age and the amount of decline in the total score of WOMAC, a very strong negative correlation was found between BMI and the amount of decline in the WOMAC pain score, and a strong negative correlation was found between the length of time passing between the two surgical procedures and the amount of decrease in WOMAC pain score.

In the literature, some studies showed that various problems occur with the tibial insert after the TKA. However, as the components are probably not well-aligned and/or well-fixed, revision total knee prosthesis appears to have been mostly preferred for treatment purposes. Isolated tibial insert exchange has been performed in the treatment of several problems, such as loosening, melting, or fracture of the tibial insert after the TKA, yet the number of studies assessing the results is limited.

In one of these studies, for example, Babis et al. performed 56 isolated tibial insert exchange procedures in 55 patients between 1985 and 1997 and followed the patients for an average of 4.6 years after revision. The mean follow-up period of the patients after index arthroplasty was 8.3 years, and their Knee Society pain and function scores, which had been on average 56 and 50.9 points before the revision, increased to 76 and 59 points in the final follow-up. In total, 25% of the knees needed a re-revision only three years after the tibial insert exchange. Of the 27 knees with preoperative instability, eight required a re-revision, and four were considered failures due to the patients’ severe pain. Of the 24 knees that were treated with the index revision because of the wear of the insert, five underwent re-revision, and one patient had to receive amputation over the knee due to chronic osteomyelitis developed in the ipsilateral ankle after the TKA [12]. Furthermore, Babis et al. also performed isolated tibial insert exchange procedures in seven patients with marked stiffness after the TKA between 1992 and 1998 and followed the patients for an average of 4.2 years after the insert exchange. The preoperative mean pain and function scores of 44 and 36.4 according to the Knee Society were then reported as 39.6 and 46 in the final follow-up. A need for a re-revision developed due to infection in one of the two knees along with aseptic loosening of the components in the other. The remaining five knees were found to be painful and stiff at the last follow-up. Of these five knees, four were reported to be severely painful, with one knee being moderately and occasionally painful [13].

In our study, the mean follow-up period after isolated tibial insert exchange was calculated as 414 (min: 182; max: 1246) days, and the average length of time until the emergence of the need for a revision after primary TKA was 6.03 years. The patients’ postoperative median WOMAC pain, stiffness, function, total scores, and VAS improved significantly. In one patient, the insert broke again only seven months after the isolated tibial insert exchange procedure, necessitating a revision TKA, and this patient was considered a failure. Unlike Babis et al.’s study [13], none of our cases developed stiffness after the primary TKA.

As another example, Willson et al. retrospectively reviewed 42 knees in 42 patients who underwent an isolated tibial polyethylene insert exchange (ITPIE) procedure for post-TKA instability, stiffness, or aseptic effusion. The intraoperative results of the study showed that all patients had well-aligned and well-fixed components. In the same study, the minimum follow-up period was two years (mean: 5.6 years; range: 2-11 years). The patients were assessed using the Knee Society rating system, upon which 12 patients (29%) underwent a subsequent ITPIE revision, with the mean length of revision being three years. Despite the improvement in mean Knee Society scores, nine (30%) of the 30 patients with no revision procedure had persistent pain during the follow-up. The study argued that the ITPIE should be performed with care even in well-defined and narrow indications. In addition, it was also concluded that “the longer the initial components performed successfully before ITPIE, the greater the likelihood of success after the ITPIE” [14].

In our study, the average follow-up period after the isolated tibial insert exchange procedures was 414 (min: 182; max: 1246) days. It was similarly determined intraoperatively that the components were well-aligned and well-fixed in all patients. Unlike Willson et al.’s study, the patients in our study were assessed using WOMAC and VAS scales in the preoperative stage and the postoperative final follow-up. Moreover, one of the knees later underwent a revision TKA in our study, accounting for 14.29%, which is considered half the rate of Willson et al.’s study; however, it would be appropriate to objectively state that our follow-up period was also shorter than that of Willson et al.’s study. In our study, the length of time until a revision after primary TKA was 6.03 years, which is twice as long as the one reported in Willson et al.’s study. Except for one knee of a patient whose WOMAC and VAS scores deteriorated and who underwent a re-revision procedure, it was observed that all patients had negligible and minimal pain during the last follow-up after the isolated tibial insert exchange procedure. Willson et al. also correlated the length of time passing from an index TKA to an ITPIE with the outcome, concluding that an ITPIE required within three years from the index TKA was 3.8 times more likely to cause a patient to undergo a revision than an ITPIE lasting longer than three years. None of our cases underwent a revision procedure for an isolated tibial insert exchange three years after the primary TKA. In contrast, a strong negative correlation was found between the length of time between two operations and the amount of decline in the WOMAC pain score in our study.

Furthermore, Tetreault et al. assessed 270 cases undergoing an isolated tibial insert exchange between 1985 and 2016 with indications of instability (55%), polyethylene wear (39%), insert breakage/separation (5%), or stiffness (1%), and they reported the survivorship rate as 68% within a 10 years’ time, without the need for any re-revision and 74% without any need for a re-revision for insert wear again in 10 years. However, it was found that re-revisions were more frequent for diagnoses other than abrasions. Average Knee Society 5 of 7 scores increased from 54 (0-94) preoperatively to 77 (38-94) within a decade [15]. In our study, the insert was broken in four knees of four patients, in addition to the loss of concave form due to insert melting/wear in three knees of two patients, and instability developed in seven knees as a result. Since the mean length of the follow-up period after isolated tibial insert exchange was 414 (min: 182; max: 1246) days in our study, it was unfortunately not possible to calculate the 10-year survivorship by relating it to the indications. However, we can clearly state that the reason for our only case of a revision procedure was a broken insert.

Baker et al. found that the mean revision age was 68 years and the mean revision time was 80 months in patients who underwent an isolated tibial insert exchange after a primary TKA and were followed up on for at least two years. The patients’ current scores (Oxford Knee Score, University of California Activity Index score, WOMAC, and Short Form 12) were compared with preoperative scores in 14 knees. Four patients (9%) subsequently underwent a revision procedure, after which significant improvement was seen in the Oxford Knee Score, the Short Form 12 physical components, and all WOMAC areas. However, according to the global WOMAC score, only 58% of patients achieved a clinically successful outcome [16]. The mean follow-up period after isolated tibial insert exchange was 414 (min: 182; max: 1246) days; in addition to that, the median revision age was 70.5 years, and the mean revision time was 72.57 months. In our study of seven knees of six patients, a statistically significant improvement was observed in the median WOMAC pain, stiffness, function, and total scores as well as in the median VAS. However, one knee needed re-revision, which was considered a failure; therefore, it can be concluded that 85.71% of the patients achieved a clinically successful result according to the total WOMAC score in our study.

In a study by Alexander et al. examining 364 patients (390 knees) treated with the ITPIE due to instability and/or polyethylene wear after TKA between 1997 and 2019, the mean age was 66.8 years, the mean BMI was 33.8 kg/m^2^, and 59% of the patients were female. The mean follow-up was 5.9 years. The Knee Society Clinical score increased from 55 preoperatively to 76 postoperatively (p < 0.001). Thirty knees (7.7%) required a revision surgery procedure in the same study (15 cases due to instability, seven aseptic loosening, three infections, two patellofemoral maltracking, one patellar fracture, one metal allergy, and one elsewhere for unknown reason) [17]. In our study, the median age of our patients was 70.5 years, and the median BMI was 30.15 kg/m^2^ with 50% of them being female. Unlike other studies in the literature, the decrease in WOMAC scores in our study was associated with age, BMI, and the length of time passing between the primary TKA and the isolated tibial insert exchange. Moreover, a strong negative correlation was found between age and the amount of decline in the WOMAC total score. There is also a very strong negative correlation between BMI and the amount of decline in the WOMAC pain score and a strong negative correlation between the length of time passing between two operations and the amount of decline in the WOMAC pain score. Research shows that isolated tibial insert exchange has been performed in the treatment of problems such as loosening, melting/wear, or breakage of the tibial insert after the TKA, yet the number of studies assessing the results is limited. From this standpoint, we believe that our study will contribute to the literature, which may be one of its strengths. Another advantage is that, unlike other studies, the WOMAC osteoarthritis index and VAS were used to assess patients before isolated tibial insert exchange and during the final follow-up.

Limitations

The most significant limitations of our study are the small sample size and the relatively short follow-up period after isolated tibial insert exchange. The number of patients who have well-aligned and well-fixed components and undergo only an isolated tibial insert degeneration is generally quite low after the primary TKA. Therefore, the number of cases with isolated tibial insert exchange was insufficient. Other limitations were the retrospective and single-center design of the study as well as the lack of comparative data, such as a control group with or without a different intervention.

## Conclusions

The isolated tibial insert exchange may be preferred in suitable patients if the components are well-aligned and well-fixed, as was the case in the early-mid-term successful results in our study. The age and BMI of the patients as well as the time since primary TKA can all be used to identify patients who are candidates for isolated tibial insert exchange.

However, we believe that prospective and multicenter studies with larger sample sizes, longer follow-up periods, and comparison groups would be beneficial to better understand the efficacy and durability of isolated tibial insert exchange as a treatment option for TKA patients experiencing tibial insert fracture and/or melting.
